# Accurate Diels-Alder Energies and *Endo* Selectivity in Ionic Liquids Using the OPLS-VSIL Force Field

**DOI:** 10.3390/ijms21041190

**Published:** 2020-02-11

**Authors:** Caroline Velez, Brian Doherty, Orlando Acevedo

**Affiliations:** Department of Chemistry, University of Miami, Coral Gables, FL 33146, USA; cxv384@miami.edu (C.V.); bwd223@nyu.edu (B.D.)

**Keywords:** ionic liquids, Diels-Alder, OPLS, QM/MM, Force Field, FEP, Monte Carlo

## Abstract

Our recently developed optimized potentials for liquid simulations-virtual site ionic liquid (OPLS-VSIL) force field has been shown to provide accurate bulk phase properties and local ion-ion interactions for a wide variety of imidazolium-based ionic liquids. The force field features a virtual site that offloads negative charge to inside the plane of the ring with careful attention given to hydrogen bonding interactions. In this study, the Diels-Alder reaction between cyclopentadiene and methyl acrylate was computationally investigated in the ionic liquid 1-butyl-3-methylimidazolium hexafluorophosphate, [BMIM][PF_6_], as a basis for the validation of the OPLS-VSIL to properly reproduce a reaction medium environment. Mixed ab initio quantum mechanics and molecular mechanics (QM/MM) calculations coupled to free energy perturbation and Monte Carlo sampling (FEP/MC) that utilized M06-2X/6-31G(d) and OPLS-VSIL gave activation free energy barriers of 14.9 and 16.0 kcal/mol for the *endo-cis* and *exo-cis* Diels-Alder reaction pathways, respectively (exptl. Δ*H*^‡^ of 14.6 kcal/mol). The *endo* selectivity trend was correctly predicted with a calculated 73% *endo* preference. The rate and selectivity enhancements present in the *endo* conformation were found to arise from preferential hydrogen bonding with the exposed C4 ring hydrogen on the BMIM cation. Weaker electronic stabilization of the *exo* transition state was predicted. For comparison, our earlier ±0.8 charge-scaled OPLS-2009IL force field also yielded a Δ*G*^‡^ of 14.9 kcal/mol for the favorable *endo* reaction pathway but did not adequately capture the highly organized solvent interactions present between the cation and Diels-Alder transition state.

## 1. Introduction

Ionic liquids are massively customizable solvents [1,2,3,4,5] that can provide significant advantages in a wide range of technological applications [6,7,8,9,10,11,12,13,14]. Composed exclusively of ions, these molten salts are often liquid at room temperature due in large part to the molecular asymmetry built into the cation, for example, 1-butyl-3-methylimidazolium [BMIM], that impedes a strong charge ordering with anions [15,16,17]. The physical and chemical properties of ionic liquids are fundamentally related to their solvent structure [18,19], which in turn is largely dependent upon Coulombic forces [20,21], dispersion interactions [22,23] and ideal ion-ion hydrogen bonding geometries [24,25]. Considerable effort has been put forth by the theoretical community to better understand the nature of these intermolecular interactions within imidazolium-based ionic liquids [26,27,28,29,30,31]. A wide range of computational methodologies have been utilized from quantum mechanics (QM) and ab initio molecular dynamics (AIMD) to empirical potential-based approaches such as molecular dynamics (MD) and coarse-grained models [32]. Compromises on the accuracy and/or speed of the simulations may have been necessary depending on the method employed. For example, QM calculations on isolated ion pairs or small ion clusters allow for very high structural and energetic accuracy [33] but lack information on temperature and dynamical effects.

**Scheme 1:**
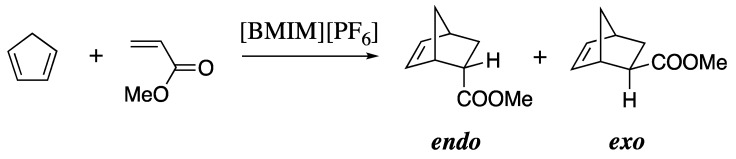
Diels-Alder reaction between cyclopentadiene and methyl acrylate in the 1-butyl-3-methylimidazolium [BMIM],[PF_6_] ionic liquid giving *endo* and *exo* bicyclic products.

Ionic liquids have drawn considerable interest as solvents for chemical reactions due to their ideal properties for organic synthesis [4] and catalysis [34]. While “ionic liquid effects” that enhance chemical reactivity can range from weak to significant, optimizing these solvent effects requires a better understanding of the contributing molecular factors [35,36,37]. Our group has provided fundamental insight through the use of on-the-fly mixed quantum mechanical and molecular mechanical (QM/MM) calculations coupled to free-energy perturbation theory and Monte Carlo sampling (FEP/MC) to simulate multiple organic reactions in ionic liquids [38], including the substitution [39], elimination [40,41], pericyclic [42,43] and rearrangement [44] reaction classes. Of particular interest here is the Diels-Alder reaction, which is known to be exceptionally sensitive to solvent effects in terms of both rates and product selectivity [45,46]. For example, Welton and coworkers reported a strong *endo* selectivity for the Diels-Alder reaction between cyclopentadiene and methyl acrylate (Scheme 1) in some of the most widely-used ionic liquids, including [BMIM][BF_4_], [BMIM][ClO_4_], [EMIM][NO_3_] and [EMIM][PF_6_] [47]. Several other research groups, including Lee [48], Kumar [49,50], Seddon [51] and Dyson [52], have also provided reports of increased *endo* product for the same cyclopentadiene and methyl acrylate reaction in multiple ionic liquid environments. While the exact origin of the enhanced selectivity is complex and dependent upon the ions chosen to construct the ionic liquid, it can be generalized to reside in the ability of imidazolium-based ionic liquids to act as a hydrogen bond donor (cation effect) moderated by its hydrogen bond accepting ability (anion effect) [53,54,55].

Molecular simulations featuring explicit solvation can provide a detailed atomistic perspective of important solute-solvent interactions [56,57,58,59]. However, accurate force field parameters may not always exist for a particular ionic liquid of interest [60,61]. In addition, critical evaluations of reported ionic liquid force fields have highlighted some common issues, for example, poor dynamics and overestimated heats of vaporization (Δ*H*_vap_) [62,63,64]. Our own optimized potentials for liquid simulations (OPLS)-based ionic liquid force field published in 2009 (i.e., OPLS-2009IL) suffered from many of these same problems [41]. A recent revisit of OPLS-2009IL tested the parameters upon an extensive and reliable set of experimental data, that included densities, heats of vaporization, viscosities, diffusion coefficients, heat capacities and surface tensions and found considerable improvement in predictions when applying a general ±0.8 charge scaling [65]. However, in order to dramatically increase the accuracy of the computed ionic liquid dynamics, thermochemical properties and local cation-anion interactions, a new force field parameterization effort was needed. Novel imidazolium-based cation parameters were created that utilized a virtual site bisecting the ring nitrogen atoms [66]. This optimized potentials for liquid simulations-virtual site ionic liquid (OPLS-VSIL) force field featured empirical charges guided by free energy of hydration calculations and new Lennard-Jones terms that were fine-tuned for the 1-alkyl-3-methylimidazolium cations and 11 different anions. The OPLS-VSIL gave quantitative reproduction of experimental data in many cases for bulk-phase solvent properties and reproduced radial distribution functions (RDFs) derived from ab initio molecular dynamics. Kappor et al. examined multiple force fields as part of a systematic benchmarking study of gas solubility in ionic liquids and reported the OPLS-VSIL to yield the most accurate Henry’s constants for CH_4_, CO_2_, NH_3_ and SO_2_ [67].

In this study, the OPLS-VSIL force field has been examined for its ability to replicate a proper reaction media environment for the chemical reaction between cyclopentadiene and methyl acrylate (Scheme 1). To investigate the reported acceleration of the *endo* product for the Diels-Alder reaction in [BMIM][PF_6_] [52], mixed ab initio QM/MM FEP/MC simulations have been performed for both the *endo*-*cis* and *exo*-*cis* reaction pathways. Reactants and transition states have been located and the computed potential free energy surfaces accurately predicted the experimental *endo* preference. The favored *endo-cis* reaction pathway was also computed using our ±0.8 charge-scaled OPLS-2009IL parameters and a comparison to the OPLS-VSIL force field is provided. This detailed investigation into the short- and long-ranged solute-solvent interactions throughout the multiple Diels-Alder reaction pathways imparts further insight into the role ionic liquids play upon this important class of organic reactions.

## 2. Methods

### 2.1. OPLS-AA Force Field

The OPLS-AA force field utilizes both intramolecular and intermolecular terms to calculate the total energy of the system [68]. The harmonic bond stretching and angle bending terms, the Fourier series for dihedral angles and the intermolecular energies from Coulomb and 12-6 Lennard-Jones terms are provided in equations 1–4. The modifiable parameters are the force constants *k*, the *r*_o_ and *θ*_o_ equilibrium bond and angle values, Fourier coefficients *V*, partial atomic charges, *q* and Lennard-Jones radii and well-depths, σ and ε.

(1) $$E_{bonds}=\underset{i}{\sum }k_{r,i}{\left(r_{i}-r_{o,i}\right)}^{2}$$

(2) $$E_{angles}=\underset{i}{\sum }k_{\theta ,i}{\left(\theta_{i}-\theta_{o,i}\right)}^{2}$$

(3) $$E_{torsions}=\underset{i}{\sum }\left[\frac{1}{2}V_{1,i}\left(1+cos\phi \right)+\frac{1}{2}V_{2,i}\left(1-cos2\phi \right)+\;\frac{1}{2}V_{3,i}\left(1+cos3\phi \right)+\;\frac{1}{2}V_{4,i}\left(1-cos4\phi \right)\right]$$

(4) $$E_{nonbond}=\underset{i}{\sum }\underset{j>i}{\sum }\left\{\frac{q_{i}q_{j}e^{2}}{r_{ij}}+4\varepsilon_{ij}\left[{\left(\frac{\sigma_{ij}}{r_{ij}}\right)}^{12}-{\left(\frac{\sigma_{ij}}{r_{ij}}\right)}^{6}\right]\right\}$$

OPLS geometric combining rules, that is, σ_ij_ = (σ_ii_σ_jj_)^1/2^ and ε_ij_ = (ε_ii_ε_jj_)^1/2^ were applied to the Lennard-Jones coefficients. Nonbonded interactions were calculated intermolecularly and for intramolecular atom pairs separated by three or more bonds with 1,4-intramolecular interactions reduced by a factor of 2.

### 2.2. QM/MM Calculations

Mixed quantum and molecular mechanical calculations were carried out using a BOSS-Gaussian interface [69] with solutes treated using the M06-2X/6-31G(d) theory level, which has been shown to yield highly accurate transition structure geometries and energies for Diels-Alder reactions [70,71]. The OPLS-VSIL and 0.8*OPLS-2009IL force fields [41,65,66] provided parameters for the [BMIM][PF_6_] ionic liquid (all parameters are available for download at https://www.github.com/orlandoacevedo/IL). Fully equilibrated binary solvent boxes containing 396 ion pairs (i.e., 198 [BMIM] cations and 198 [PF_6_] anions) for OPLS-VSIL and 376 ion pairs for 0.8*OPLS-2009IL were constructed that were periodic and tetragonal with long-range electrostatics treated using Ewald sums. Potentials of mean force (PMF) calculations coupled to Metropolis Monte Carlo statistical mechanics were utilized to compute a free-energy profile for the Diels-Alder reaction at 25 °C and 1 atm. Solutes were inserted into the ionic liquid boxes and the system was equilibrated for 28.5 million MC configurations. A final production run of 1.5 million MC configurations was carried out. QM energies and atomic charges calculations were performed using Gaussian 09 revision E.01 [72] for each attempted move of the solute, that is, every 1000 configurations. For solute-solvent electrostatic energy contributions, CM5 charges [73] were computed for the solutes with a scaling factor of 1.20 [74]. Lennard-Jones interactions between the reactants and solvent atoms were computed using OPLS parameters [68].

The simulations were carried out using the BOSS program [75]. The [BMIM] cations were fully flexible with all internal geometries sampled, whereas the [PF_6_] anion was simulated as rigid. Solute-solvent and solvent-solvent intermolecular cutoff distances of 1200 pm were employed for the tail carbon atom of each side chain (methyl and butyl), a midpoint on the butyl chain and the ring carbon between both nitrogen atoms for [BMIM]. The center atom P was used for the anion cutoff. If any distance was within the cutoff, the entire solvent-solvent interaction was included. Allowed ranges for rotations, translations and dihedral angle movements led to overall acceptance rates of ca. 30% for new configurations. The BOSS program automatically sets ranges for bond stretching and angle bending on the basis of force constants and temperature. All calculations were run on computers located at the University of Miami.

## 3. Results and Discussion

**Figure 1:**
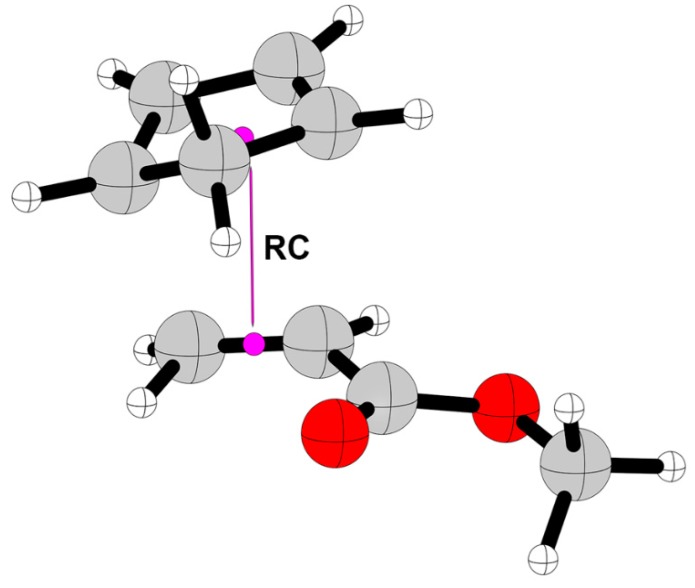
Diels-Alder reaction coordinate (RC) defined as distance between the midpoints of the C=C bond of methyl acrylate and cyclopentadiene’s terminal carbons. The *exo-cis* transition structure from a gas-phase M06-2X/6-31G(d) optimization is illustrated.

Mixed QM/MM FEP/MC calculations were performed for the reaction between cyclopentadiene and methyl acrylate in [BMIM][PF_6_] using a modified version of BOSS 4.9 with Gaussian 09, revision E.01 used to perform solute QM single-point calculations. Our prior Diels-Alder QM/MM studies utilized a semiempirical QM (SQM) method, for example, PDDG/PM3 [76], that gave over-estimated activation barriers compared to experiment [42,77,78]. In this work, the M06-2X/6-31G(d) theory level has been employed instead, as it has been shown to provide highly accurate transition structure geometries and energies for Diels-Alder reactions [70,71]. The use of density functional theory (DFT) instead of SQM significantly increased the cost of the simulations, for example, approximately 3–4 days for M06-2X/6-31G(d) compared to 10–15 min for PDDG/PM3 per 1.5 million MC configurations. The ionic liquid solvent was treated using the OPLS-VSIL or 0.8*OPLS-2009IL force fields [65,66]. Free-energy profiles were computed by perturbing a reaction coordinate (RC) defined by the distance between two dummy atoms located at the midpoint of the diene terminal carbon atoms and the midpoint of the diene C=C bond (Figure 1), as done in our previous computational investigation of the reaction in chloroaluminate ionic liquids [42]. The RC distance ranged from 204 to 400 pm with a 2 pm increment. Fifty λ-windows were used in conjunction with double-wide sampling to compute the free-energy profile covering the reactant complex and transition structure along the reaction path. Each FEP window had 28.5 and 1.5 million MC configurations of equilibration and averaging, respectively, which entailed 30,000 M06-2X/6-31G(d) single-point calculations. A total of 15 million DFT calculations were required per full reaction profile.

The QM/MM computed free energy of activation, Δ*G*^‡^, for the Diels-Alder reaction between cyclopentadiene and methyl acrylate in the ionic liquid [BMIM][PF_6_] is provided in Table 1. The *endo-cis* and *exo-cis* reaction pathways yielded Δ*G*^‡^ values of 14.9 and 16.0 kcal/mol, respectively, using OPLS-VSIL. Both transition states occurred at an RC of 216 pm. Uncertainties in the free energy barriers were derived using statistical uncertainties in each λ-window that gave an overall uncertainty in the Δ*G*^‡^ of ± 0.2 kcal/mol. The computed energies were in good agreement with an experimental Δ*H*^‡^ of 14.6 kcal/mol that was reported by Tiwari and Kumar for the same reaction in [BMIM][PF_6_] using a temperature-dependent kinetic investigation [79]. The computed ΔΔ*G*^‡^ difference of 1.1 kcal/mol corresponded to a 73% *endo* preference, which is consistent with the experimentally observed *endo* enhancement present in ionic liquids relative to conventional solvents, for example, 67% *endo* for the reaction in water (25 °C and 1 atm) [80,81] compared to 79% (i.e., 3.8:1.0 *endo:exo* ratio) for the same reaction in [BMIM][PF_6_] [52]. The favored *endo-cis* reaction pathway was recalculated using our charge-scaled 0.8*OPLS-2009IL force field and an identical Δ*G*^‡^ value of 14.9 ± 0.2 kcal/mol was obtained.

**Figure 2:**
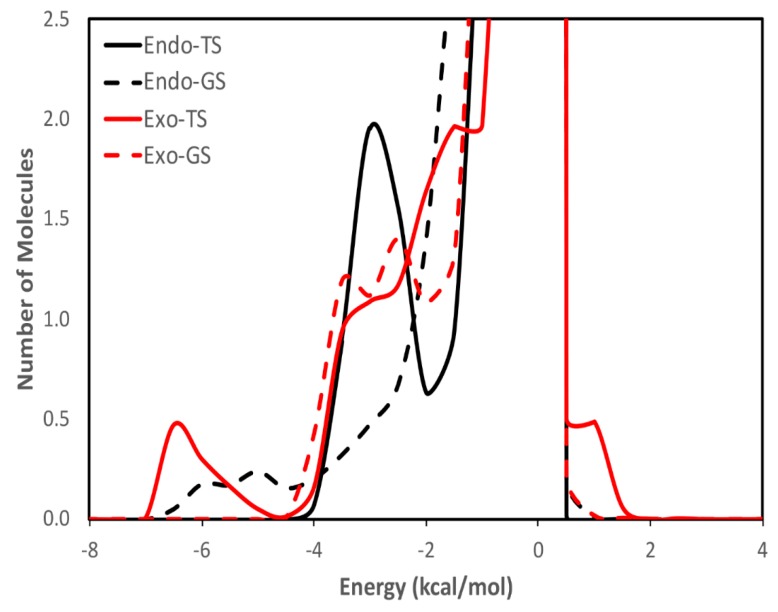
Solute-solvent energy pair distributions for the Diels-Alder reaction between cyclopentadiene and methyl acrylate for the reactants (GS) and transition state (TS) in [BMIM][PF_6_] at 25 °C using the OPLS-VSIL force field. The ordinate provides the number of solvent ions that interact with the solutes and their interaction energy on the abscissa. Units for ordinate are number of molecules per kcal/mol.

Solute-solvent energy pair distributions for the Diels-Alder *endo-cis* and *exo-cis* reaction pathways derived from the OPLS-VSIL ionic liquid environment are given in Figure 2. The interaction energies are obtained by analyzing the QM/MM/MC results at the FEP windows representative of the reactants and transition state. The distributions record the average number of ions from the [BMIM][PF_6_] that interacted with the reacting system and their corresponding energies. Highly favorable interaction energies between the solute and solvent systems are found in the left-most region and the large band at 0 kcal/mol represents the majority of the ions which are located in the outer solvent shells. From Figure 2, the *endo* and *exo* transition state plots are clearly different with the *endo* TS possessing a larger peak centered at an energy of −3 kcal/mol relative to the smaller *exo* TS peak centered at a lower energy of −5 kcal/mol. Integrating the distributions from −10.0 to −3.0 kcal/mol finds that the *endo* route increased the number of interactions by 1.1 in going from the reactants to the transition state, whereas the number of interactions increased by 0.5 in the *exo* route (Table 2). Extending the integration to −2 kcal/mol gave a similar trend where the number of ion interactions increased by 1.2 and 0.8 in going from the reactants to the transition state for the *endo* and *exo* routes, respectively (the absolute number of ions in reactants and TS are 3.9 and 5.1 for *endo* and 5.2 and 6.0 for *exo*). Solute-solvent energy pair distributions derived from the 0.8*OPLS-2009IL force field is provided in the Supporting Information Figure S1.

**Figure 3:**
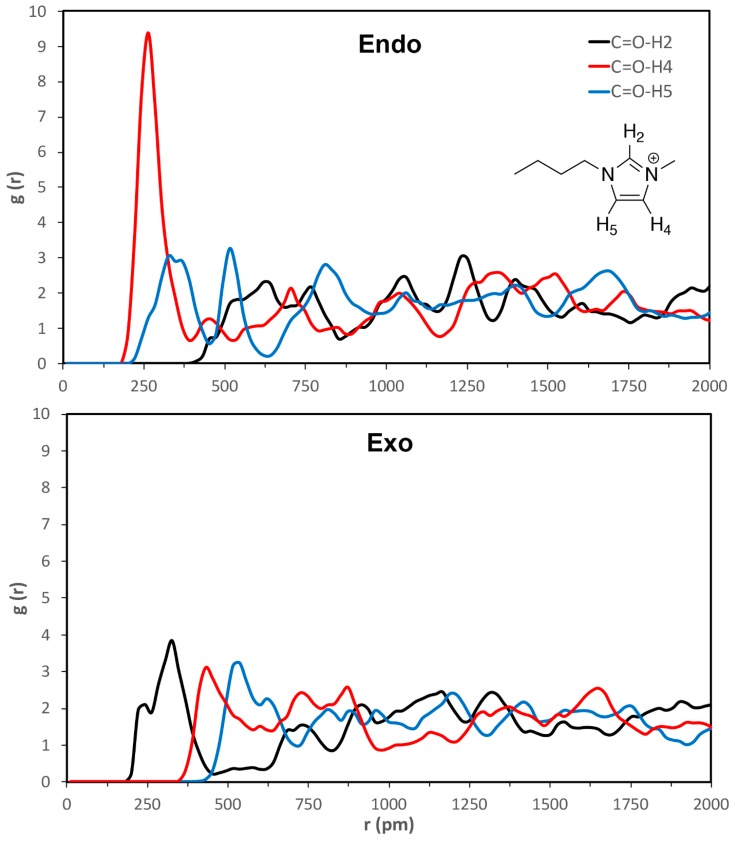
Radial distribution functions for the *endo* (top) and *exo* (bottom) transition states in the cyclopentadiene and methyl acrylate reaction in [BMIM][PF_6_] at 25 °C using the OPLS-VSIL force field between the ring protons on BMIM and carbonyl oxygen of methyl acrylate.

The solute-solvent structure for the reaction in [BMIM][PF_6_] can be further characterized by radial distribution functions, *g*(*r*). To increase the sampling of the solvent at the transition state FEP window, representative transition structures from the *endo* and *exo* reaction pathways were fixed with the solvent allowed to move for an additional 40 million MC configurations. Radial distribution functions (RDF) were computed on the resulting trajectory using the TRAVIS analyzer program [82]. Figure 3 provides the RDF plots for the *endo-cis* and *exo-cis* transition states in [BMIM][PF_6_], where the ring hydrogen atoms (H2 given in black, H4 in red and H5 in blue) interacted with the carbonyl oxygen atom of methyl acrylate. For the *endo* TS, a large radial distribution peak *g*(*r*) of 9.4 centered around 260 pm formed for the H4^…^O=C interaction. Interestingly, the most acidic proton on BMIM, that is, H2 located on the 2-position carbon atom bisecting the nitrogen atoms [83,84], had a much smaller and broader peak centered further away from the carbonyl oxygen. The H2 atom is shielded by the adjacent methyl and butyl groups and possesses a partial charge value similar to the other ring hydrogens [66]. This trend was previously reported for the same Diels-Alder reaction in 1-ethyl-3-methylimidazolium chloroaluminate melts, where hydrogen bonds were not computationally observed between the H2 atom on EMIM and the carbonyl oxygen of methyl acrylate [42]. The adjacent H5 ring atom also had a smaller peak when interacting with the carbonyl oxygen, with a peak height around 3.0 and centered at 325 pm. Alternatively, the 0.8*OPLS-2009IL force field gave relatively smaller peaks for all the *endo-cis* TS ring hydrogen atoms (H2, H4 and H5), with H5^…^O=C possessing the largest peak of g(r) = 4.5 centered around 325 pm (Supporting Information Figure S2). Similar to OPLS-VSIL, the 0.8*OPLS-2009IL had the H2^…^O=C interaction centered much further away at 577 pm with a height of 2.9 (Figure S2), also finding that the H2 atom pointed away from the methyl acrylate during the reaction and toward adjacent anions instead. Whereas the presence of hydrogen bond donors in the reaction medium leads to enhanced *endo:exo* selectivities in Diels- Alder reactions [53], higher selectivities have been observed in 1-alkyl-2,3-dimethylimidazolium ionic liquids, where the 2-position hydrogen has been replaced with a methyl group [52].

The RDF peaks for all of the ring hydrogens interacting with the carbonyl oxygen of methyl acrylate in the *exo-cis* transition structure were relatively small compared to the H4^…^O=C interaction computed in the *endo* TS conformation (Figure 3). In the case of the *exo* TS, the H2^…^O=C interaction was the most favored with the closest peak centered around 240 pm at a height of 2.1 and a second adjacent peak at around 325 pm with a *g*(*r*) of 3.8. As the cyclopentadiene ring is rotated away from the ester moiety of methyl acrylate in the *exo* TS conformation (Figure 1), sterics may play a reduced role in hindering the H2^…^O=C interaction as compared to the *endo* conformation, particularly when considering that the CM5 charges for carbonyl oxygen in the representative *endo-cis* and *exo-cis* transition structures are similar with computed values of –0.405 and –0.409 e, respectively (Table 3). Radial distribution functions of the imidazolium ring hydrogens interacting with the ester oxygen of methyl acrylate are given for both the *endo* and *exo* TS conformations using the OPLS-VSIL in the Supporting Information Figure S3. All of the ester-O^…^H2/H4/H5 peaks were small, *g*(*r*) < 4, suggesting that weaker interactions prevailed compared to the H4^…^O=C in the *endo* TS conformation. This is consistent with the less negative CM5 charges computed for the ester oxygen, that is, –0.283 and –0.285 in the representative *endo-cis* and *exo-cis* transition structures, respectively, compared to the carbonyl oxygen charges (Table 3).

**Figure 4:**
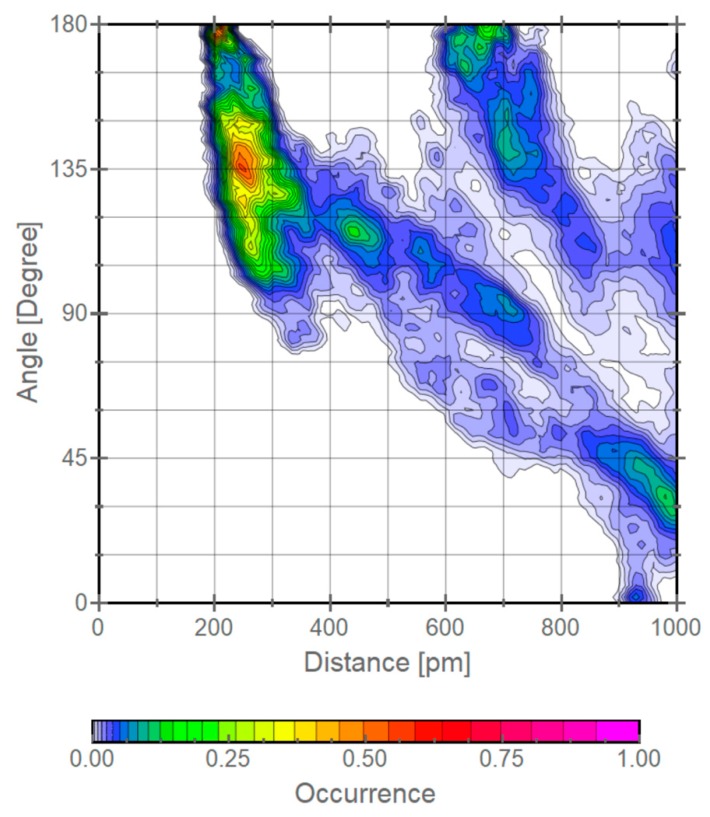
Angle between the normal to the C4 and H4 atoms on the imidazolium cation and the line connecting the carbonyl O atom of methyl acrylate as a function of the distance between BMIM and the substrate *endo-cis* transition structure for the Diels-Alder reaction in [BMIM][PF_6_] using the OPLS-VSIL.

Combined distribution functions (CDF) have been used to monitor the angle between a reference normal vector from a BMIM ring carbon atom (C2, C4 or C5), the corresponding covalently bonded ring hydrogen (H2, H4 or H5 respectively) and the carbonyl oxygen atom on methyl acrylate as a function of the distance between the cation and Diels-Alder transition structure. The same 40 million MC trajectories from the RDF calculations were utilized. Figure 4 provides the OPLS-VSIL CDF plot for the C4–H4^…^O=C interaction in the *endo* TS conformation. The greatest number of interaction occurrences were computed at cation-substrate distances between 200 and 260 pm, which is consistent with the large *endo* TS peak centered around 260 in the RDF analysis (Figure 3). An idealized hydrogen bond angle of 180 degrees was found at approximately 200 pm. A second strong occurrence was found at a distance of around 250 pm with an approximate angle of 135 degrees. Hunt and coworkers previously performed MD simulations on pure [EMIM][Cl] and [BMIM][Cl] ionic liquids and found that hydrogen bonding in these molten salts is highly angle dependent and temperature differences result in a variable, not static, network of hydrogen bonds [29,85]. The CDF plots for all ring hydrogens (H2, H4 and H5) interacting with the carbonyl oxygen atom of the *endo* and *exo* transition states are given in the Supporting Information for both the OPLS-VSIL and 0.8*OPLS-2009IL force fields (Figures S4–S6). The OPLS-VSIL CDF plots showed that major occurrences between the ring hydrogens and carbonyl oxygen were consistent with the larger peaks at distances found in the RDF plots and had idealized hydrogen bonding angles of 110–180 degrees [86]. However, the 0.8*OPLS-2009IL force field yielded multiple ring hydrogen-carbonyl oxygen occurrences at distances varying from 250–400 pm for the H4 and H5 atoms with angles that impeded strong hydrogen bonding, that is, 50–110 degrees (Figure S6). The OPLS-VSIL force field was parameterized with careful attention given to hydrogen bonding interactions between the ions themselves [66] and it appears that for this particular Diels-Alder reaction the solute-solvent intermolecular interactions are better organized as compared to the 0.8*OPLS-2009IL.

**Figure 5:**
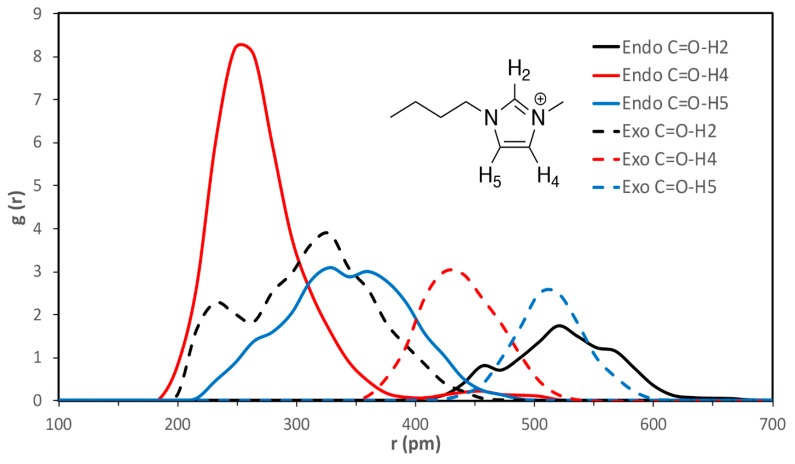
Nearest neighbor distribution for the *endo-cis* (solid lines) and *exo-cis* (dashed lines) Diels-Alder transition structures in [BMIM][PF_6_] between the ring protons on BMIM (H2, H4 and H5) and the carbonyl oxygen of methyl acrylate using the OPLS-VSIL.

To further investigate the solute-solvent interactions, nearest neighbor distributions were calculated for the *endo-cis* and *exo-cis* transition states. These distributions track the distance between the closest ion (cation or anion) and the substrate over each 40 million MC step trajectory derived from the OPLS-VSIL and 0.8*OPLS-2009IL force fields. In particular, the distances between the hydrogen atoms on the imidazolium ring (H2, H4 and H5) and the oxygens on methyl acrylate, that is, carbonyl and ester, were monitored and provided in Table 4. A tight distance of 260 ± 30 pm between C=O^…^H4 was calculated for the *endo* TS trajectory using OPLS-VSIL, while longer distances were computed for C=O^…^H2 and C=O^…^H5, that is, 520 ± 40 and 330 ± 50 pm, respectively. The average distances followed a trend of C=O^…^H4 < C=O^…^H5 < C=O^…^H2 for the *endo* TS conformation that was consistent with the computed RDF plot, which reported the C=O^…^H4 interaction to possess the largest g(r) height and closest peak center distance (Figure 3). However, the *exo* OPLS-VSIL system followed a different trend where the average distances were C=O^…^H2 < C=O^…^H4 < C=O^…^H5. Again, the *exo* TS nearest neighbor distributions were in sync with the RDF plot, which showed the C=O^…^H2 interaction to have the closest peak center distance compared to H4 and H5. Despite C=O^…^H2 being potentially the strongest hydrogen bond interaction found between BMIM and the *exo* transition state, the greater distance of 310 ± 60 pm for C=O^…^H2 compared to 260 ± 30 pm for C=O^…^H4 in the *endo* TS, suggests that BMIM may provide less electrostatic stabilization of the transition state in the *exo-cis* reaction pathway. Figure 5 shows a nearest neighbor distribution plot highlighting the distances between the BMIM ring and the methyl acrylate substrate at the transition state FEP windows. Analysis of the *endo* TS trajectory computed with the 0.8*OPLS-2009IL force field found that the H4 and H5 ring hydrogens preferred to interact with the carbonyl oxygen at distances of 340 ± 70 and 320 ± 50 pm, respectively, (Table 4 and Supporting Information Figure S7). Both interactions were further in distance than those computed by OPLS-VSIL, which suggests that the 0.8*OPLS-2009IL force field parameters may provide weaker solute-solvent intermolecular interactions.

## 4. Conclusions

In summary, mixed QM/MM FEP/MC calculations utilizing M06-2X/6-31G(d) and the OPLS-VSIL force field were carried out for the Diels-Alder reaction between cyclopentadiene and methyl acrylate in [BMIM][PF_6_]. Accurately modeling room temperature ionic liquids can be a particularly challenging endeavor when employing nonpolarizable force field parameters [56,60]. Fortunately, the recently developed OPLS-VSIL force field has been shown to yield improved bulk phase properties, local ion-ion interactions and gas solubility predictions for imidazolium-based ionic liquids compared to earlier force fields [66,67]. However, the OPLS-VSIL potentials have never been tested as a reaction medium for chemical reactions. The Diels-Alder reaction studied here represents a significant challenge to model as the system is known to be exceptionally sensitive to ionic liquids in terms of both rates and product selectivity [47]. The computed *endo*-*cis* and *exo*-*cis* reaction pathways yielded accurate Δ*G*^‡^ values of 14.9 ± 0.2 kcal/mol and 16.0 ± 0.2 kcal/mol, respectively (exptl. Δ*H*^‡^ of 14.6 kcal/mol [79]). This represents a significant improvement over our previous semiempirical PDDG/PM3-based QM/MM FEP/MC method that gave overestimated Δ*G*^‡^ values of 40–43 kcal/mol for the same reaction in the chloroaluminate ionic liquids [EMIM][AlCl_4_] and [EMIM][Al_2_Cl_7_] [42]. In addition, the OPLS-VSIL correctly predicted the experimentally preferred *endo* selectivity with a computed *endo*% of 73% in [BMIM][PF_6_] (exptl. 79% [52]).

The exact origin of enhanced Diels-Alder *endo* selectivities derived from ionic liquids is complex but can be generalized to reside in the ability of imidazolium-based ionic liquids to act as a hydrogen bond donor (cation effect) moderated by its hydrogen bond accepting ability (anion effect) [53,54,55]. A detailed investigation into the short- and long-ranged solute-solvent interactions throughout both the *endo* and *exo* Diels-Alder reaction pathways was provided through the analysis of solute-solvent energy pair distributions, radial distribution functions, combined distribution functions and nearest neighbor distributions. The rate and selectivity enhancements present in the *endo* conformation arise from preferential hydrogen bonding with the exposed C4 ring hydrogen on the BMIM cation, which was absent in the *exo* conformation. Utilizing our earlier ±0.8 charge-scaled OPLS-2009IL force field to compute the favorable *endo* reaction pathway yielded an accurate Δ*G*^‡^ of 14.9 ± 0.2 kcal/mol but did not adequately capture the highly organized solvent structure present between the cation and substrate. The OPLS-VSIL force field was parameterized with careful attention given to hydrogen bonding interactions between ions in pure ionic liquids. The well-organized solute-solvent intermolecular interactions predicted for this particular Diels-Alder reaction by the OPLS-VSIL indicates it may also provide an accurate description of reaction medium effects.

## Figures and Tables

**Table 1:** Free Energy of Activation, Δ*G*^‡^ (kcal/mol), at 25 °C for the Diels-Alder Reaction Between Cyclopentadiene and Methyl Acrylate in [BMIM][PF_6_] Computed Using M06-2X/OPLS-VSIL free energy perturbation and Monte Carlo sampling (FEP/MC).

calc. *endo* ^a^	calc. *exo*	exptl. ^b^	calc. *endo*%	exptl. *endo*% ^c^
14.9 ± 0.2	16.0 ± 0.2	14.6	73	79

^a^ M06-2X/0.8*OPLS-2009IL FEP/MC also gave 14.9 ± 0.2 kcal/mol. ^b^ Δ*H*^‡^ obtained from transition-state theory plot [79]. ^c^ Reference [52].

**Table 2:** Number of Solute-Solvent Interactions for the Reactants and Transition State Integrating from −10.0 to −3.0 kcal/mol from quantum mechanics and molecular mechanics (QM/MM) FEP/MC Simulations.

	Reactants	Transition State
*endo*	1.8	2.9
*exo*	2.7	3.2

**Table 3:** CM5 Charges for the Carbonyl and Ester Oxygen Atoms on Methyl Acrylate for the representative *endo-cis* and *exo-cis* Diels-Alder transition structures using M06-2X/6-31G(d).

	carbonyl O	ester O
*endo-cis* TS	−0.405	−0.283
*exo-cis* TS	−0.409	−0.285

**Table 4:** Average Distance (pm) Calculated Using a Nearest Neighbor Distribution Between the BMIM Ring Hydrogens (H2, H4 and H5) and Carbonyl Oxygen (C=O) or Ester Oxygen (esterO) from the Diels-Alder *Endo-Cis* and *Exo-Cis* Transition Structures in [BMIM][PF_6_] using OPLS-VSIL and 0.8*OPLS-2009IL.

	C=O-H2	C=O-H4	C=O-H5	esterO-H2	esterO-H4	esterO-H5
VSIL_*endo*TS	520 ± 40	260 ± 30	330 ± 50	640 ± 20	380 ± 80	430 ± 40
VSIL_*exo*TS	310 ± 60	440 ± 30	510 ± 20	360 ± 50	580 ± 30	540 ± 60
2009_*endo*TS	550 ± 30	340 ± 70	320 ± 50	450 ± 30	470 ± 80	360 ± 40

## Supplementary Materials

Supplementary materials can be found at https://www.mdpi.com/1422-0067/21/4/1190/s1. Additional solute-solvent energy pair distributions; radial distribution functions; combined distribution functions; nearest neighbor distributions; and the complete reference for Gaussian 09.
