# Supplementary material for: Accurate Diels-Alder Energies and Endo Selectivity in Ionic Liquids Using the OPLS-VSIL Force Field
Source: Int J Mol Sci. 2020 Feb 11;21(4):1190. doi: 10.3390/ijms21041190 (PMC7072795; doi:10.3390/ijms21041190)
Supplement: Supplementary file 1 [file ijms-21-01190-s001.pdf]

Accurate Diels-Alder Energies and *Endo* Selectivity in Ionic Liquids using the OPLS-VSIL Force Field

Caroline Velez, Brian Doherty, and Orlando Acevedo*

Department of Chemistry, University of Miami, Coral Gables, Florida 33146

E-mail: orlando.acevedo@miami.edu

Supporting Material.

Figure S1. Solute-solvent energy pair distributions for 0.8*OPLS-2009IL……………..………….……………2

Figure S2. Radial distribution functions for *endo* TS using 0.8*OPLS-2009IL…………..…………..………..3

Figure S3. Radial distribution functions for ester-O using OPLS-VSIL ……………………….………………4

Figure S4. Combined distribution functions for *endo* TS using OPLS-VSIL ……………….…………………5

Figure S5. Combined distribution functions for *exo* TS using OPLS-VSIL ……………….……….…………6

Figure S6. Combined distribution functions for *endo* TS using 0.8*OPLS-2009IL ……….…………………7

Figure S7. Nearest neighbor distributions for *endo* TS using 0.8*OPLS-2009IL ………….…………………8

Gaussian 09 E.01 Reference…………..…………………………………………..…………….……………8

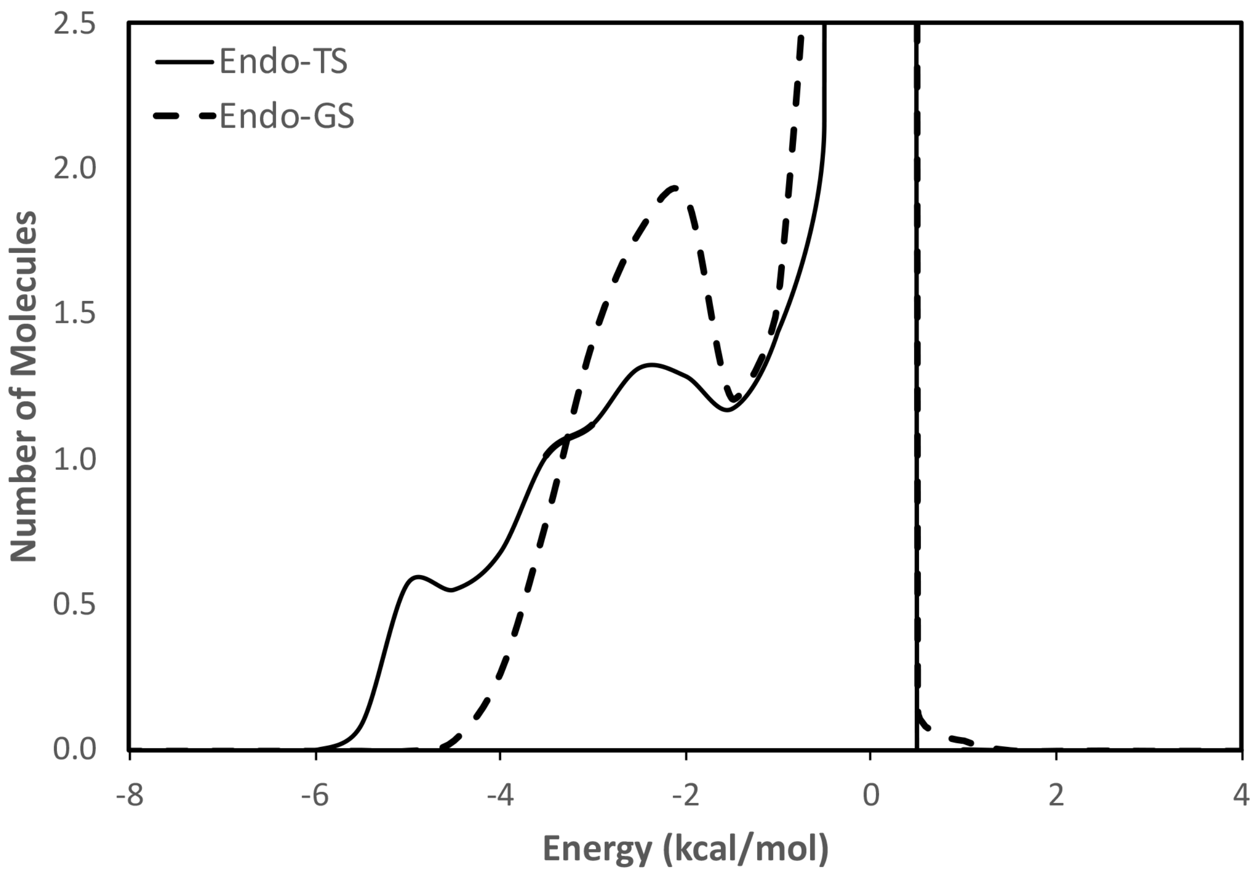

Figure S1. Solute-solvent energy pair distributions for the Diels-Alder reaction between cyclopentadiene and methyl acrylate for the reactants (GS) and transition state (TS) in [BMIM][PF_6] at 25 °C using the 0.8*OPLS-2009IL force field. The ordinate provides the number of solvent ions that interact with the solutes and their interaction energy on the abscissa. Units for ordinate are number of molecules per kcal/mol.

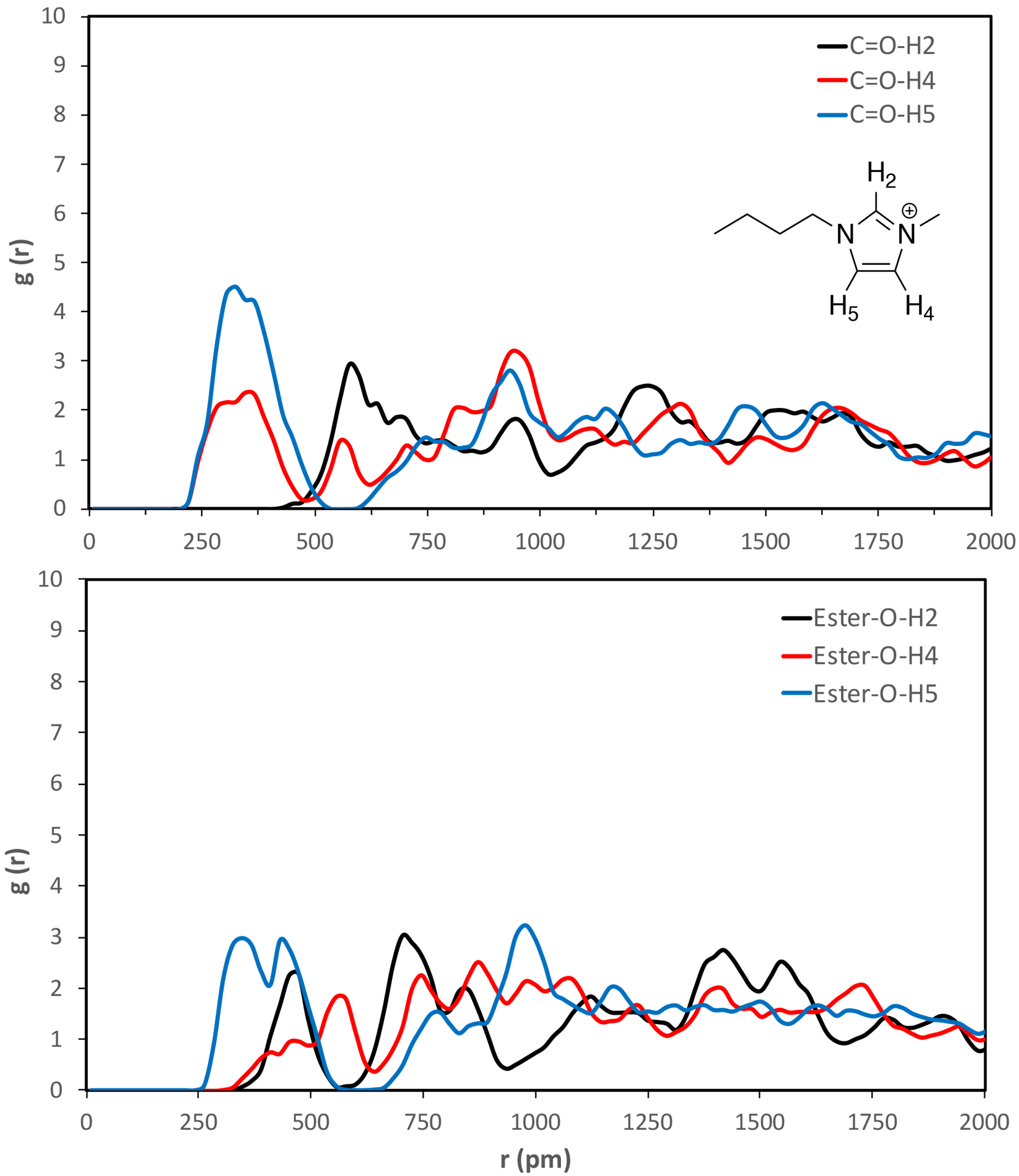

Figure S2. Radial distribution functions for the *endo* transition state for the cyclopentadiene and methyl acrylate reaction in [BMIM][PF_6] at 25°C using the 0.8*OPLS-2009IL force field between the ring protons on BMIM and carbonyl (top) and ester (bottom) oxygens of methyl acrylate.

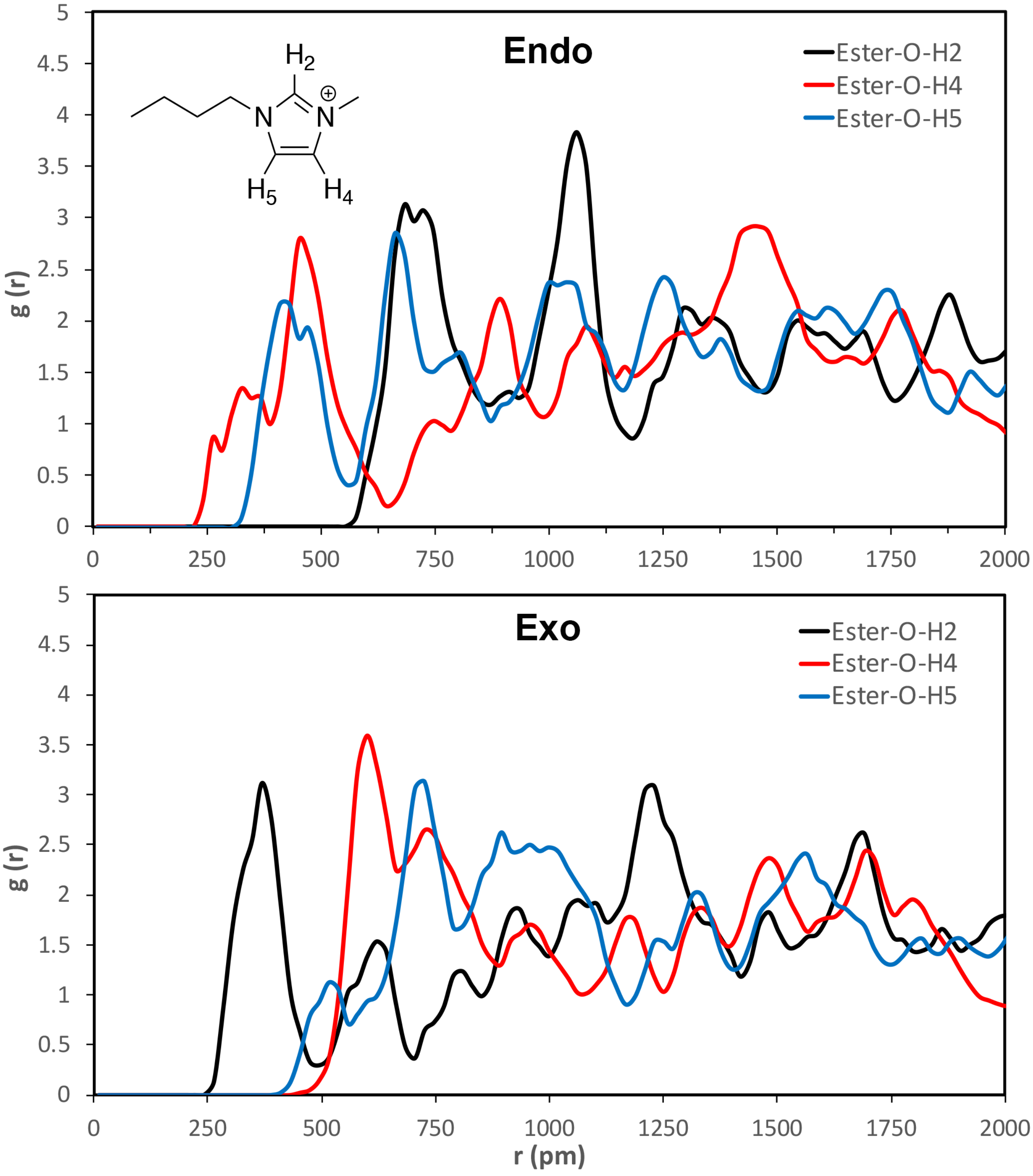

Figure S3. Radial distribution functions for the *endo* (top) and *exo* (bottom) transition states in the cyclopentadiene and methyl acrylate reaction in [BMIM][PF_6] at 25°C using the OPLS-VSIL force field between the ring protons on BMIM and ester oxygen of methyl acrylate.

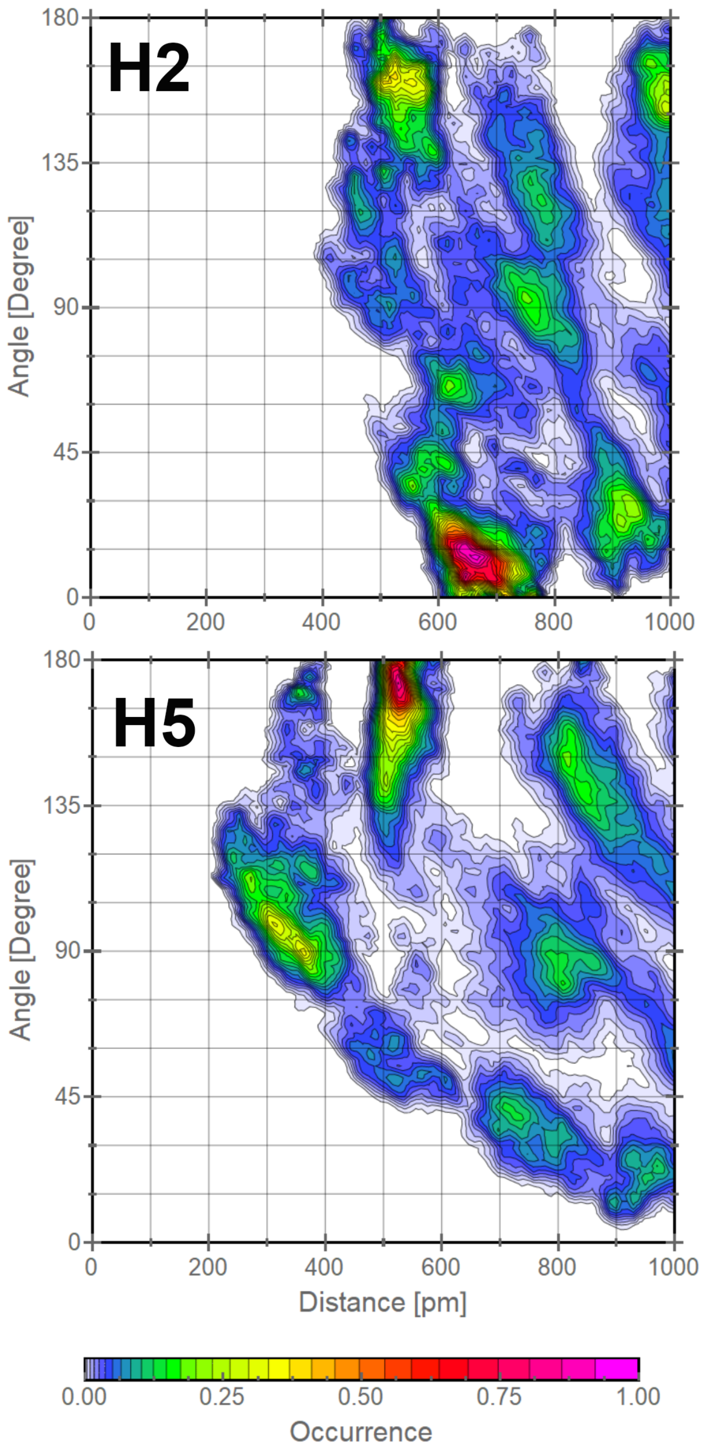

Figure S4. Angle between the normal to the C and H2 and H5 atoms on the imidazolium cation and the line connecting the carbonyl O atom of methyl acrylate as a function of the distance between BMIM and the substrate *endo-cis* transition structure for the Diels-Alder reaction in [BMIM][PF_6] using the OPLS-VSIL.

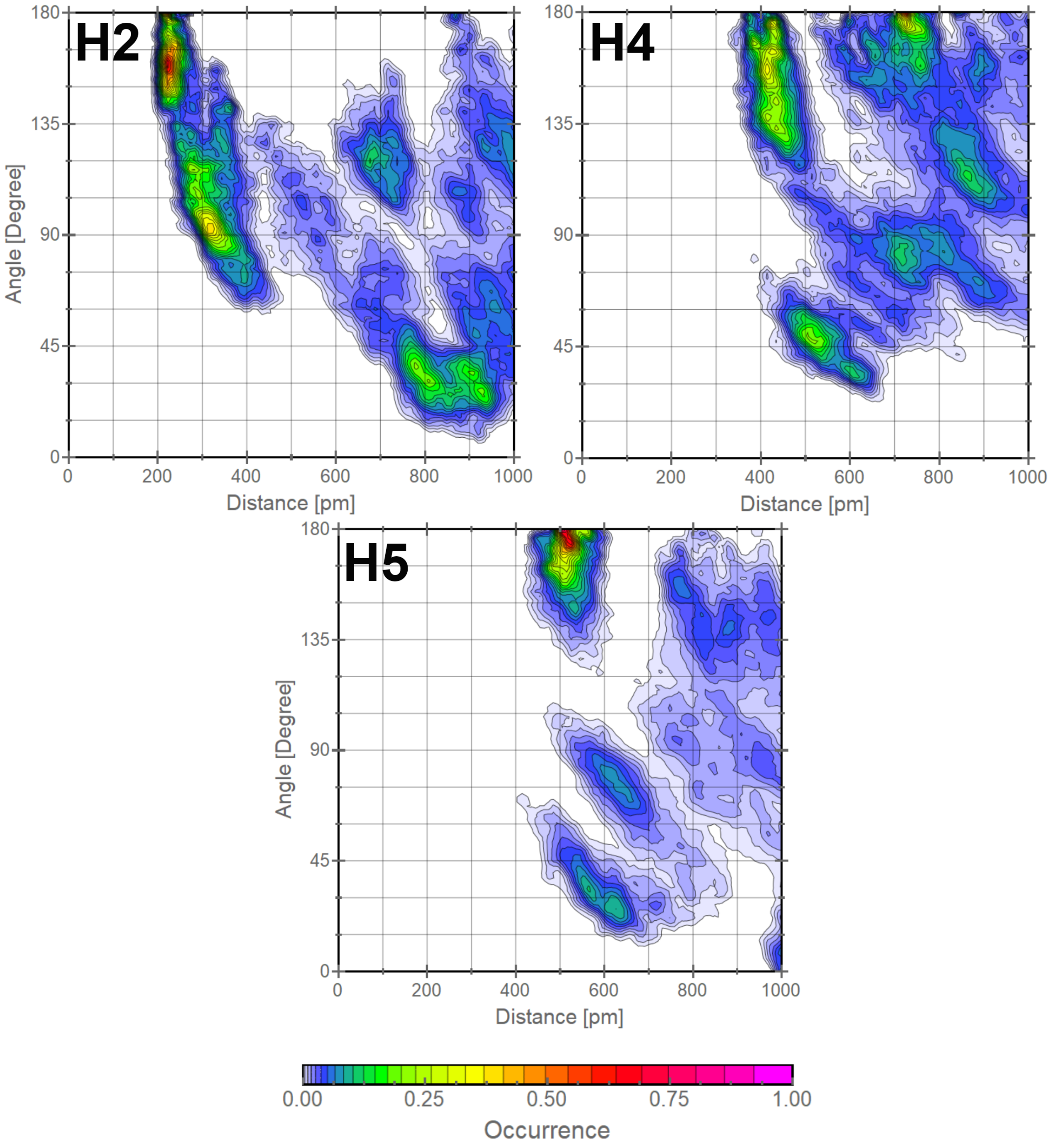

Figure S5. Angle between the normal to the C and H2, H4, and H5 atoms on the imidazolium cation and the line connecting the carbonyl O atom of methyl acrylate as a function of the distance between BMIM and the substrate *exo-cis* transition structure for the Diels-Alder reaction in [BMIM][PF_6] using the OPLS-VSIL.

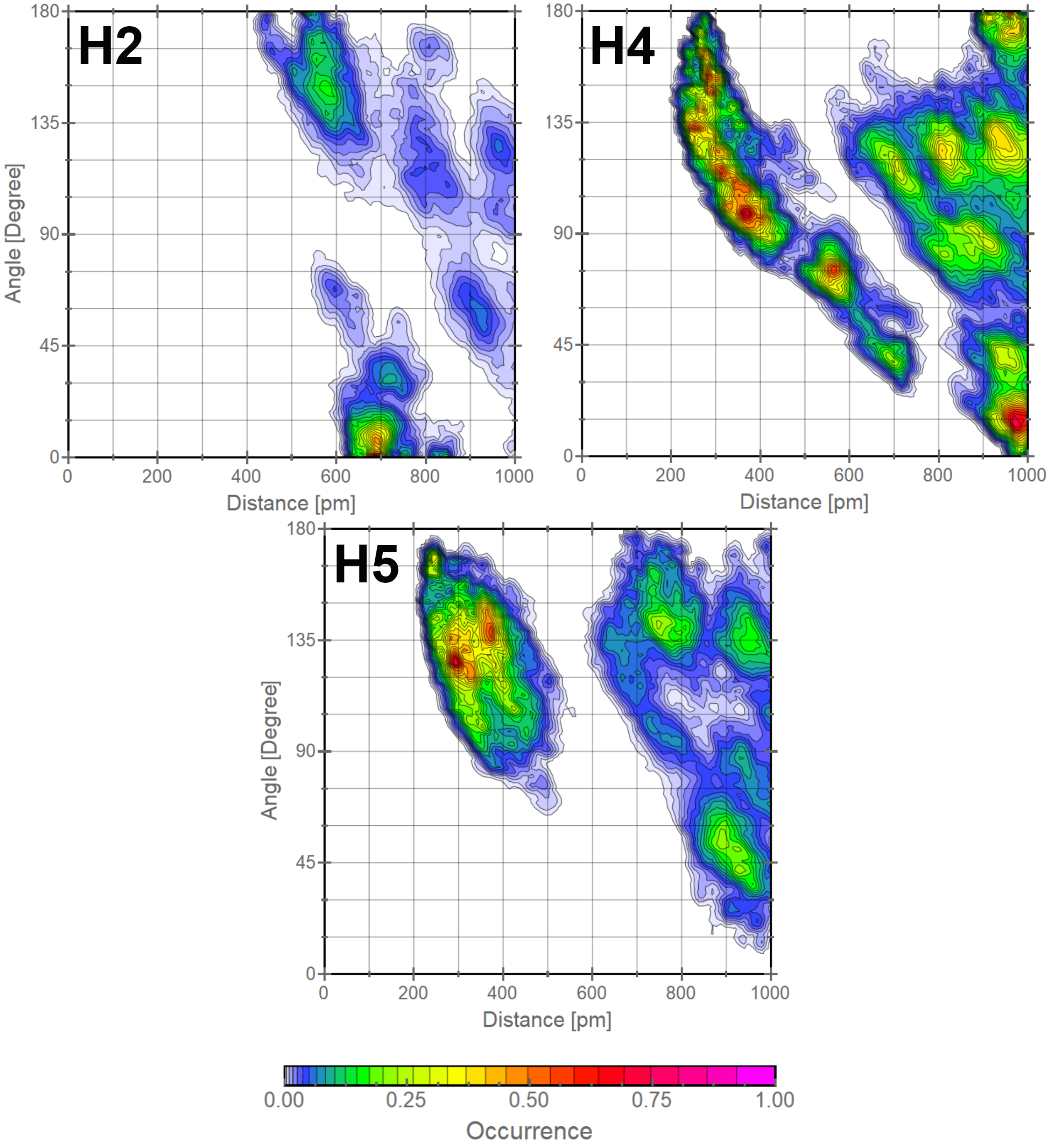

Figure S6. Angle between the normal to the C and H2, H4, and H5 atoms on the imidazolium cation and the line connecting the carbonyl O atom of methyl acrylate as a function of the distance between BMIM and the substrate *endo-cis* transition structure for the Diels-Alder reaction in [BMIM][PF_6] using the 0.8*OPLS-2009IL.

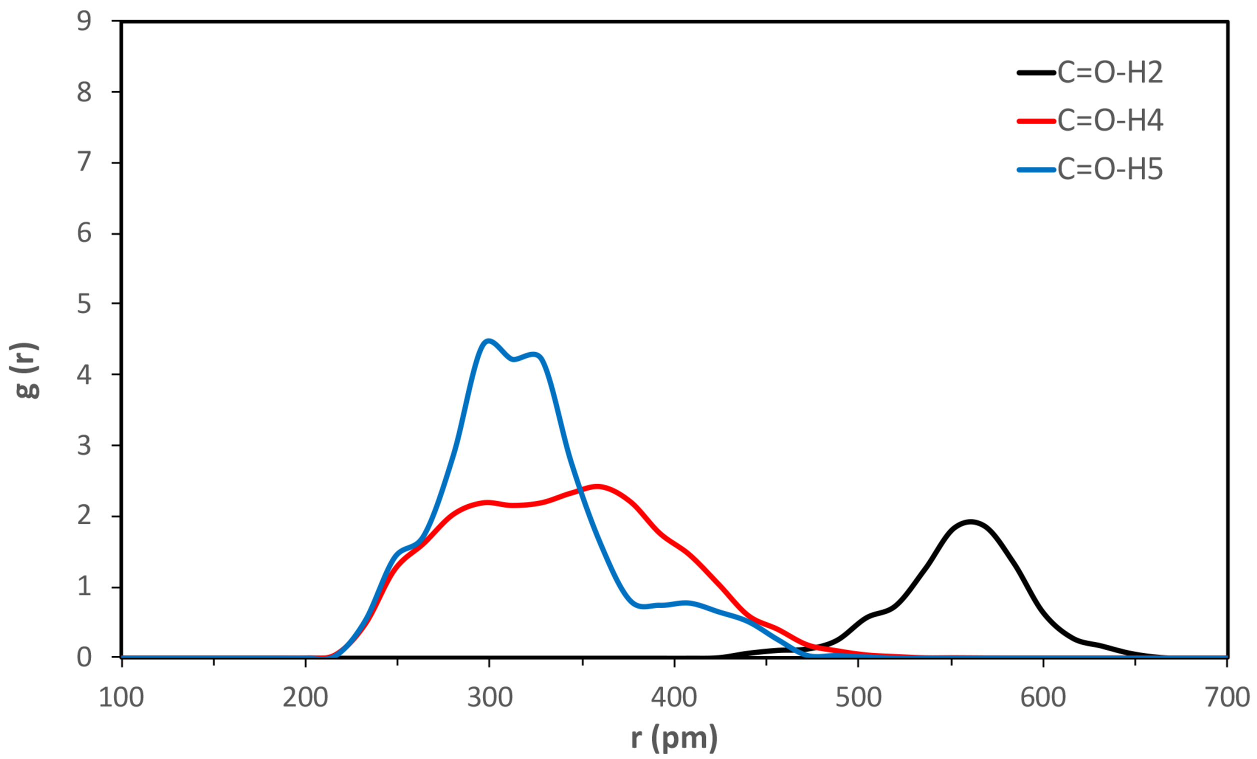

Figure S7. Nearest neighbor distribution for the *endo-cis* Diels-Alder transition structure in [BMIM][PF_6] between the ring protons on BMIM (H2, H4, and H5) and the carbonyl oxygen of methyl acrylate using the 0.8*OPLS-2009IL.

Complete Gaussian 09, E.01 citation

Gaussian 09, Revision E.01, Frisch, M. J.; Trucks, G. W.; Schlegel, H. B.; Scuseria, G. E.; Robb, M. A.; Cheeseman, J. R.; Scalmani, G.; Barone, V.; Mennucci, B.; Petersson, G. A.; Nakatsuji, H.; Caricato, M.; Li, X.; Hratchian, H. P.; Izmaylov, A. F.; Bloino, J.; Zheng, G.; Sonnenberg, J. L.; Hada, M.; Ehara, M.; Toyota, K.; Fukuda, R.; Hasegawa, J.; Ishida, M.; Nakajima, T.; Honda, Y.; Kitao, O.; Nakai, H.; Vreven, T.; Montgomery, J. A., Jr.; Peralta, J. E.; Ogliaro, F.; Bearpark, M.; Heyd, J. J.; Brothers, E.; Kudin, K. N.; Staroverov, V. N.; Kobayashi, R.; Normand, J.; Raghavachari, K.; Rendell, A.; Burant, J. C.; Iyengar, S. S.; Tomasi, J.; Cossi, M.; Rega, N.; Millam, J. M.; Klene, M.; Knox, J. E.; Cross, J. B.; Bakken, V.; Adamo, C.; Jaramillo, J.; Gomperts, R.; Stratmann, R. E.; Yazyev, O.; Austin, A. J.; Cammi, R.; Pomelli, C.; Ochterski, J. W.; Martin, R. L.; Morokuma, K.; Zakrzewski, V. G.; Voth, G. A.; Salvador, P.; Dannenberg, J. J.; Dapprich, S.; Daniels, A. D.; Farkas, Ö.; Foresman, J. B.; Ortiz, J. V.; Cioslowski, J.; Fox, D. J. Gaussian, Inc., Wallingford CT, 2009.
